# Supplementary material for: Ongoing shuffling of protein fragments diversifies core viral functions linked to interactions with bacterial hosts
Source: Nat Commun. 2023 Nov 28;14:7460. doi: 10.1038/s41467-023-43236-9 (PMC10684548; doi:10.1038/s41467-023-43236-9)
Supplement: Supplementary file 3 — Description of Additional Supplementary Files [file 41467_2023_43236_MOESM3_ESM.pdf]

Description of Additional Supplementary Files

File Name: Supplementary Data S1
Description: Mapping of original PHROG annotations (columns 1 and 2) to alternative, simplified names (columns 5 and 6). Columns 3 and 4 provide the total number of sequences and PHROGs per original functional annotation.

File Name: Supplementary Data S2
Description: List of odds ratios and p-values for each ECOD domain tested (Fisher's one-tailed exact test) for being over-represented in mosaic rHMMs (as shown in Figure 2C and 2D). P-values were adjusted using Bonferroni correction for multiple testing.

File Name: Supplementary Data S3
Description: List of odds ratios and p-values for each functional class tested (Fisher's one-tailed exact test) for being over-represented in mosaic rHMMs (as shown in Figure 4). P-values were adjusted using Bonferroni correction for multiple testing.
