# Supplementary material for: Ongoing shuffling of protein fragments diversifies core viral functions linked to interactions with bacterial hosts
Source: Nat Commun. 2023 Nov 28;14:7460. doi: 10.1038/s41467-023-43236-9 (PMC10684548; doi:10.1038/s41467-023-43236-9)
Supplement: Supplementary file 7 — Reporting Summary [file 41467_2023_43236_MOESM7_ESM.pdf]

nature portfolio

Corresponding author(s): Rafal Mostowy
Last updated by author(s): Oct 20, 2023

Reporting Summary

Nature Portfolio wishes to improve the reproducibility of the work that we publish. This form provides structure for consistency and transparency in reporting. For further information on Nature Portfolio policies, see our Editorial Policies and the Editorial Policy Checklist.

Statistics

For all statistical analyses, confirm that the following items are present in the figure legend, table legend, main text, or Methods section.

n/a	Confirmed	
☐	☒	The exact sample size (*n*) for each experimental group/condition, given as a discrete number and unit of measurement
☒	☐	A statement on whether measurements were taken from distinct samples or whether the same sample was measured repeatedly
☐	☒	The statistical test(s) used AND whether they are one- or two-sided<br>*Only common tests should be described solely by name; describe more complex techniques in the Methods section.*
☒	☐	A description of all covariates tested
☐	☒	A description of any assumptions or corrections, such as tests of normality and adjustment for multiple comparisons
☐	☒	A full description of the statistical parameters including central tendency (e.g. means) or other basic estimates (e.g. regression coefficient) AND variation (e.g. standard deviation) or associated estimates of uncertainty (e.g. confidence intervals)
☐	☒	For null hypothesis testing, the test statistic (e.g. *F*, *t*, *r*) with confidence intervals, effect sizes, degrees of freedom and *P* value noted<br>*Give P values as exact values whenever suitable.*
☒	☐	For Bayesian analysis, information on the choice of priors and Markov chain Monte Carlo settings
☒	☐	For hierarchical and complex designs, identification of the appropriate level for tests and full reporting of outcomes
☒	☐	Estimates of effect sizes (e.g. Cohen's *d*, Pearson's *r*), indicating how they were calculated

Our web collection on statistics for biologists contains articles on many of the points above.

Software and code

Policy information about availability of computer code

Data collection	No software was used specifically for data collection. Downloaded datasets include:<br>NCBI RefSeq (release 209)<br>UniClust30 (release UniRef30_2020_06_hhsuite).<br>PHROGs (version 4)<br>ECOD (version ECOD_F70_20200207)
Data analysis	We used two custom code pipelines for data analysis. The first, "phage-protein-modularity-data," processes NCBI RefSeq data, carries out HMM profile construction, and performs all-by-all HMM comparison as well as HMM-HMM comparison to PHROGs and ECOD databases. The second pipeline, "phage-protein-modularity-figures," takes the output of the first pipeline and generates all outputs and figures presented in the publication. Both pipelines are available on GitHub under their respective release versions:<br><br>https://github.com/bioinf-mcb/phage-protein-modularity-data, archived under https://doi.org/10.5281/zenodo.10021838<br>https://github.com/bioinf-mcb/phage-protein-modularity-figures, archived under https://doi.org/10.5281/zenodo.10026778

For manuscripts utilizing custom algorithms or software that are central to the research but not yet described in published literature, software must be made available to editors and reviewers. We strongly encourage code deposition in a community repository (e.g. GitHub). See the Nature Portfolio guidelines for submitting code & software for further information.

Data

Policy information about availability of data

All manuscripts must include a data availability statement. This statement should provide the following information, where applicable:

- Accession codes, unique identifiers, or web links for publicly available datasets
- A description of any restrictions on data availability
- For clinical datasets or third party data, please ensure that the statement adheres to our policy

Input data necessary to reproduce the results have been uploaded to Figshare and are accessible under the following link: https://doi.org/10.6084/m9.figshare.24004092. Source data are provided in the paper.

Research involving human participants, their data, or biological material

Policy information about studies with human participants or human data. See also policy information about sex, gender (identity/presentation), and sexual orientation and race, ethnicity and racism.

Reporting on sex and gender	This study was focused on studying genomes of bacterial viruses from a public database, hence sex and gender information do not apply.
Reporting on race, ethnicity, or other socially relevant groupings	N/A
Population characteristics	N/A
Recruitment	N/A
Ethics oversight	N/A

Note that full information on the approval of the study protocol must also be provided in the manuscript.

Field-specific reporting

Please select the one below that is the best fit for your research. If you are not sure, read the appropriate sections before making your selection.

☐ Life sciences ☐ Behavioural & social sciences ☒ Ecological, evolutionary & environmental sciences

For a reference copy of the document with all sections, see nature.com/documents/nr-reporting-summary-flat.pdf

Ecological, evolutionary & environmental sciences study design

All studies must disclose on these points even when the disclosure is negative.

Study description	The study is a computational analysis focused on understanding the evolutionary genomics of bacterial viruses (bacteriophages) with an emphasis on protein modularity and domain mosaicism. We analysed 4,548 complete bacteriophage genomes from NCBI Virus, resulting in 462,721 predicted protein sequences. These were clustered into 133,624 groups using mmseqs2. Hidden Markov Model (HMM) profiles were constructed for each cluster, leading to 133,574 representative HMMs (rHMMs). These rHMMs were pairwise compared using hhblits to assess sequence and structural similarities. Functional annotation was performed using the PHROGs database. Domain detection was carried out using the ECOD database. The study is hierarchical in design, moving from genome-level data to protein clusters, and finally to domain-level information. There are no experimental units or replicates as the study is entirely computational.
Research sample	The research sample consists of 4,548 complete bacteriophage genomes obtained from NCBI Virus, representing a comprehensive set of bacteriophage diversity. These genomes yielded 462,721 predicted protein sequences, clustered into 133,624 groups. The sample aims to represent the broad spectrum of bacteriophage genomic and proteomic diversity. The choice of complete genomes ensures high-quality data for robust computational analysis. No organism taxa, sex, or age range is applicable as the study focuses on viral genomes.
Sampling strategy	The sampling procedure involved downloading all complete bacteriophage genomes from NCBI Virus that met specific criteria, resulting in 4,548 genomes. The sample size was determined by the availability of complete bacteriophage genomes in the database. Given the computational nature of the study and the broad representation of bacteriophage diversity, the sample size is considered sufficient for robust statistical and computational analyses.
Data collection	Data collection was automated and involved downloading complete bacteriophage genomes from the NCBI Virus database using specific criteria. Open reading frames were then detected using the MultiPhate2 approach, and protein sequences were clustered with mmseqs2. All procedures were executed in a computational environment, eliminating the need for manual data recording. The data collection and processing were carried out by the research team as part of the study's computational pipeline.

Timing and spatial scale	Data collection occurred in a single batch on January 2022. Given the computational nature of the study, periodicity and frequency of sampling were not applicable. The rationale for this one-time data collection was to obtain a snapshot of complete bacteriophage genomes available in the NCBI Virus database at that time. The data is global in scope, encompassing bacteriophage genomes from various sources without geographical limitation. There were no gaps between collection periods.
Data exclusions	Data exclusions were made based on specific criteria. Representative protein sequences with more than 10 unknown characters were excluded from further analysis. This was done to ensure the quality and reliability of the Hidden Markov Model (HMM) profiles generated. These exclusion criteria were pre-established to maintain the integrity of the dataset for computational analysis.
Reproducibility	The study is computational in nature and relies on well-documented algorithms and publicly available databases. The code for data analysis is scripted in a manner that allows for easy replication of the results and shared with the publication. All parameters used in the algorithms, such as Hidden Markov Model (HMM) profile construction and sequence clustering, are explicitly stated. This ensures that the study can be reproduced by other researchers.
Randomization	In this computational study, the allocation into groups is based on the inherent characteristics of the bacteriophage genomes and their derived protein sequences. The study does not involve random allocation or control of covariates as it would in a traditional experimental design. Instead, sequences are grouped based on computational analyses, such as clustering based on sequence similarity and functional annotation. Therefore, the concept of random allocation or control of covariates is not applicable to this study.
Blinding	Blinding was not relevant to this computational study. The analyses were performed on bacteriophage genomes and their derived protein sequences using automated computational methods. There was no subjective interpretation of data that could be influenced by knowledge of the sample identities, making the concept of blinding inapplicable in this context. All steps are clearly outlined and can be reproduced for verification.

Did the study involve field work? ☐ Yes ☒ No

Reporting for specific materials, systems and methods

We require information from authors about some types of materials, experimental systems and methods used in many studies. Here, indicate whether each material, system or method listed is relevant to your study. If you are not sure if a list item applies to your research, read the appropriate section before selecting a response.

Materials & experimental systems

n/a	Involved in the study	
☒	☐	Antibodies
☒	☐	Eukaryotic cell lines
☒	☐	Palaeontology and archaeology
☒	☐	Animals and other organisms
☒	☐	Clinical data
☒	☐	Dual use research of concern
☒	☐	Plants

Methods

n/a	Involved in the study	
☒	☐	ChIP-seq
☒	☐	Flow cytometry
☒	☐	MRI-based neuroimaging
